# Trends in heart failure costs for commercially insured patients in the United States (2006–2021)

**DOI:** 10.1186/s12913-024-11240-4

**Published:** 2024-07-08

**Authors:** Jianwei Zheng, Islam Abudayyeh, Cyril Rakovski, Louis Ehwerhemuepha, Ahmad Rezaie Mianroodi, Jay N Patel, Alomari Ihab, Chizobam Ani

**Affiliations:** 1 Department of Preventive and Social Medicine Internal Medicine Department, Charles R Drew University of Medicine and Science, 1731 E. 120th St, Los Angeles, CA USA; 2 Schmid College of Science and Technology, Chapman University, Orange, CA USA; 3 Loma Linda Veteran Administration Healthcare, Loma Linda, CA USA; 4 Internal Medicine Department, Charles R Drew University of Medicine and Science, Los Angeles, CA USA; 5 Children’s Hospital of Orange County, Orange, CA USA; 6 School of Medicine, University of California, Irvine, CA USA; 7 Internal Medicine Department, UCLA, Los Angeles, CA USA

**Keywords:** Heart failure, Medical costs, Commercially insured, Healthcare disparities, Real-world data, And heat failure management

## Abstract

**Background:**

Although prior research has estimated the overarching cost burden of heart failure (HF), a thorough analysis examining medical expense differences and trends, specifically among commercially insured patients with heart failure, is still lacking. Thus, the study aims to examine historical trends and differences in medical costs for commercially insured heart failure patients in the United States from 2006 to 2021.

**Methods:**

A population-based, cross-sectional analysis of medical and pharmacy claims data (IQVIA PharMetrics^®^ Plus for Academic) from 2006 to 2021 was conducted. The cohort included adult patients (age > = 18) who were enrolled in commercial insurance plans and had healthcare encounters with a primary diagnosis of HF. The primary outcome measures were the average total annual payment per patient and per cost categories encompassing hospitalization, surgery, emergency department (ED) visits, outpatient care, post-discharge care, and medications. The sub-group measures included systolic, diastolic, and systolic combined with diastolic, age, gender, comorbidity, regions, states, insurance payment, and self-payment.

**Results:**

The study included 422,289 commercially insured heart failure (HF) patients in the U.S. evaluated from 2006 to 2021. The average total annual cost per patient decreased overall from $9,636.99 to $8,201.89, with an average annual percentage change (AAPC) of -1.11% (95% CI: -2% to -0.26%). Hospitalization and medication costs decreased with an AAPC of -1.99% (95% CI: -3.25% to -0.8%) and − 3.1% (95% CI: -6.86–0.69%). On the other hand, post-discharge, outpatient, ED visit, and surgery costs increased by an AAPC of 0.84% (95% CI: 0.12–1.49%), 4.31% (95% CI: 1.03–7.63%), 7.21% (95% CI: 6.44–8.12%), and 9.36% (95% CI: 8.61–10.19%).

**Conclusions:**

The study’s findings reveal a rising trend in average total annual payments per patient from 2006 to 2015, followed by a subsequent decrease from 2016 to 2021. This decrease was attributed to the decline in average patient costs within the Medicare Cost insurance category after 2016, coinciding with the implementation of the Medicare Access and CHIP Reauthorization Act (MACRA) of 2015. Additionally, expenses related to surgical procedures, emergency department (ED) visits, and outpatient care have shown substantial growth over time. Moreover, significant differences across various variables have been identified.

**Supplementary Information:**

The online version contains supplementary material available at 10.1186/s12913-024-11240-4.

## Background

Heart failure (HF) is a clinical syndrome resulting from structural or functional cardiac disorders that impair ventricular function [1]. In the USA, HF affects an estimated 6.9 million people [2], and its prevalence is expected to increase by 24% to nearly 8.5 million by 2030 [3]. An analysis, using data from the large US registry linked to Medicare data, reported 5-year all-cause readmission, readmission for HF, and mortality rates of 80.4%, 42.3%, and 75.4%, respectively [4]. Moreover, the economic burden of HF on healthcare systems is considerable and expected to increase as the prevalence of HF grows [5]. In the USA, the total cost of care (direct and indirect costs) for HF in 2020 is estimated at $43.6 billion, with over 70% of costs attributed to medical costs [3]. Without improvements in outcomes, the annual total cost of care in the USA is projected to increase to $69.7 billion by 2030 [3].

While studies have estimated the overall cost burden of HF [3, 4, 6], a comprehensive analysis examining historical trends and differences in medical expenses specifically for commercially insured patients using real-world data remains scarce. Focusing on commercial insurance claim data is crucial to understanding HF costs for patients below 65 years old, as Medicare insurance plans do not cover most of this population. Therefore, this work aims to fill this gap by thoroughly examining historical trends and differences in medical expenses for commercially insured HF patients.

## Methods

### Data acquisition

We conducted this analysis using the IQVIA PharMetrics^®^ Plus for Academic database, which is a longitudinal database of medical and pharmacy claims data from over 110 million unique patients across the United States. Patient mix is primarily Commercial preferred provider organization (PPO) & health maintenance organization (HMO). PharMetrics Plus for Academic involves patient-level data acquisition rather than hospital-level data. The data encompasses patients with specific insurance providers, including commercial, Managed Medicaid, Medicare Risk (also known as Medicare Advantage), Medicare Cost (Medicare Supplemental), and self-insured individuals. Enrollees in PharMetrics Plus for Academic display similar distributions of geographic location and population age as outlined in US Census data (shown in supplemental material Fig. 1), suggesting that the data from PharMetrics Plus for Academic can potentially serve as a representative sample reflecting broader national trends in terms of both geographical and age-related demographics.

### Inclusion criteria

We created a cohort of beneficiaries enrolled in commercial insurance plans for a continuous year from 2006 to 2021. Enrollment inclusion criteria ensured that insurance claims submitted for all healthcare encounters for these patients were available. In this cohort, we classified patients as having HF if they had a healthcare encounter with an International Classification of Diseases (ICD) code 9/10 for HF. The ICD 9 and 10 Clinical Modification (CM) codes for HF are listed in supplemental material eTable 1. With this approach, we aimed to gather comprehensive information on all potential inpatient and outpatient care encounters associated with HF, a methodology employed in prior studies utilizing claims data [7]. As ICD-10 codes in administrative data have continued to shift since mandatory adoption in 2015, ICD 9 CM codes are converted to ICD 10 CM codes accordingly for heterogeneity analysis.

### Identify relevant cost components and inflation adjustment

Begin by identifying all potential cost components associated with HF management, including total annual medical costs, hospitalization costs, post-discharge costs, outpatient care costs (including total outpatient care and emergency visit costs), medication costs, and surgery costs over the 16-year study period. Thus, we used the Bureau of Labor Statistics (BLS) Consumer Price Index (CPI) for medical care [8] to account for inflation. The costs recorded in each claim were adjusted to 2021 US dollars. Outpatient cost refers to the expenses associated with medical services or procedures that are provided to patients who are not admitted to a hospital or healthcare facility overnight.

### Cost aggregation

Aggregate the costs across all relevant resources to obtain the total cost of HF management for each patient. This involves summing up the costs associated with hospitalizations, outpatient visits, medications, and other healthcare services incurred by each patient over each year.

### Assessing comorbidities using the Charlson comorbidity index

To evaluate the influence of comorbidities on costs, we calculated the Charlson Comorbidity Index (CCI) [9] for each patient annually. The CCI assigns a weighted score to different comorbidities based on their impact on mortality, with higher scores signifying a more significant burden of comorbidities and a higher risk of mortality.

### Subgroup analyses

Subgroup analyses were conducted according to age group, gender, insurance type, US region, state, comorbidity index, and HF sub-phenotype. Regarding age groups, the population was classified as < 50 years, 50–54 years, 55–59 years, 60–64 years, 65–69 years, 70–74 years, 75–79 years, 80–84 years, and > = 85 years. The insurance types included Commercial, Managed Medicaid insurance, Medicare Risk (presently known as Medicare Advantage), Medicare Cost (Medicare Supplemental), and Self-Insured insurance. According to the ICD codes listed in primary diagnosis, the HF sub-phenotypes include systolic, diastolic, and combined systolic and diastolic HF.

### Statistical analyses

Patient characteristics were described using appropriate measures such as percentages, means with standard deviations, or medians. Joinpoint regression program analyses [10] were used to identify the best-fitting points (years) with a statistically significant increase or decrease in trends from 2006 to 2021 for each variable and age group. Final models were selected using the Grid Search Method to determine the optimal number of joinpoints. The empirical quantile method was introduced to construct confidence intervals. We report the annual percent change (APC) with 95% CIs for each trend line segment. The average annual percent changes (AAPCs) with 95% CIs are reported for the entire period (2006–2021). All analyses were conducted using R 3.5 [11] and Python 3.8.

## Results

### Study population characteristics

Table 1 summarizes the baseline characteristics of 422,289 HF patients (male 49.45%) over 16 years, with a notable proportion (37.53%) aged 85 and above and 70.27% aged 65 and above. Congestive HF was the primary diagnosis for 95.57% of patients. Commercial insurance and Medicare Cost covered the largest proportions of patients at 36.76% and 44.42%, respectively. Geographically, the Mid-West region had the highest percentage of patients with HF, followed by the Eastern, Southern, and Western regions. A higher rate of patients had an index of 5 or more (34.43%), indicating a higher burden of comorbidities.

**Table 1:** Demographic information and clinical characteristics for HF patients at enrollment. Systolic HF includes cases with ICD codes starting with I502, diastolic HF with I503, and combined systolic and diastolic HF with I504

	Number of Patients
Total number of patients	419,273
Male (n, %)	207,119 (49.4)
**Age group (n, %)**	
>=85	177,476 (42.33)
80–84	7,697 (1.84)
75–79	21,755 (5.19)
70–74	37,158 (8.86)
65–69	45,429 (10.84)
60–64	42,911 (10.23)
55–59	32,999 (7.87)
50–54	22,318 (5.32)
45–49	13,547 (3.23)
40–44	7,796 (1.86)
35–39	4,478 (1.07)
30–34	2,733 (0.65)
25–29	1,551 (0.37)
20–24	1,080 (0.26)
< 19	345 (0.08)
>=65	289,515 (69.05)
< 65	129,758 (30.95)
Congestive HF (n, %)	400,719 (95.57)
Systolic HF (n, %)	124,096 (29.6)
Diastolic HF (n, %)	133,821 (31.92)
Combined Systolic and Diastolic HF (n, %)	51,674 (12.32)
**Payment type (n, %)**	
Commercial	153,413 (36.59)
Managed Medicaid	21,202 (5.06)
Medicare Risk (presently known as Medicare Advantage)	100,699 (24.02)
Medicare Cost (Medicare Supplemental)	121,677 (29.12)
Self-Insured	22,282 (5.31)
**Region (n, %)**	
East	100,586 (23.99)
Middle West	121,703 (29.13)
South	101,214 (24.14)
West	95,770 (22.84)
**Comorbidity Index Group (n, %)**	
1	71,711 (17.1)
2	69,950 (16.68)
3	70,213 (16.75)
4	56,340 (13.44)
>=5	151,059 (36.03)

### Cost categories and hospitalization rate

**Fig. 1:**
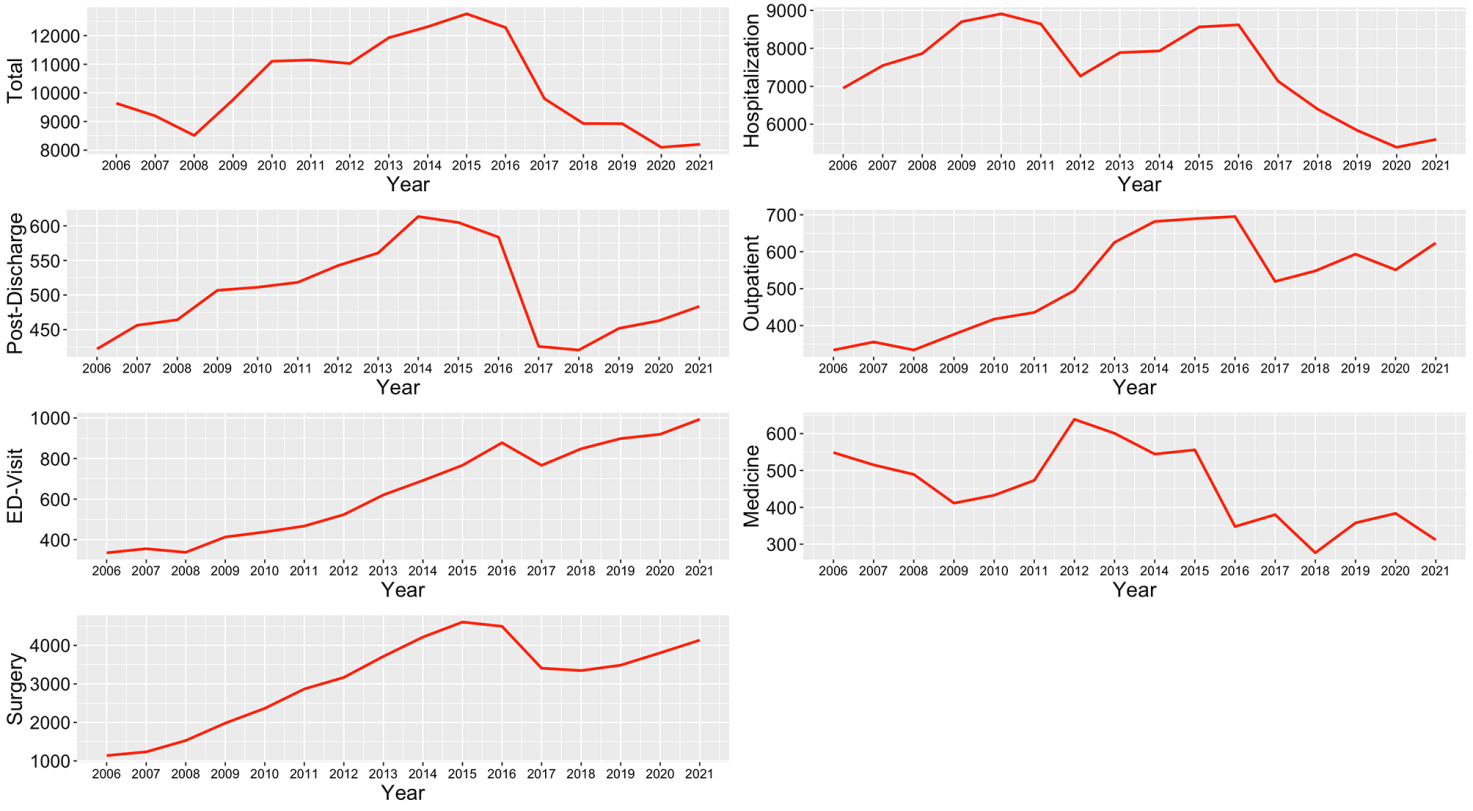
Annual trends in average cost per patient for hospitalization, outpatient, post-discharge, ED visit, surgery, and medicine from 2006 to 2021

The average total yearly payment per patient increased from $9,636.99 in 2006 to $12,763.90 in 2015, with an annual percentage change (APC) of 3.82% (95% CI: 2.26–5.75%), before dropping to $8,201.89 in 2021 with an APC of -8.05% (95% CI: -11.44% to -5.52%) (shown in Table 2; Fig. 1). From 2006 to 2021, hospitalization and medication costs decreased with an AAPC of -1.99% (95% CI: -3.25% to -0.8%) and − 3.1% (95% CI: -6.86–0.69%). Post-discharge, outpatient, ED visit, and surgery costs increased by an AAPC of 0.84% (95% CI: 0.12–1.49%), 4.31% (95% CI: 1.03–7.63%), 7.21% (95% CI: 6.44–8.12%), and 9.36% (95% CI: 8.61–10.19%) (shown in Table 2; Fig. 1). The annual cost breakdown by categories is presented in the supplemental material eTable 2.

**Table 2:** Trends in subcategories and subgroups 2006–2021

			AAPC (95% CI) ^a^		APC and joinpoint segments	
	2006	2021	2006–2021	2017–2021	Trend years	APC (95% CI)
Cost Category						
Total	$9,636.99	$8,201.89	-1.1 (-2.00 to -0.26) *	-8.05 (-11.44 to -5.52) *	2006–2015	3.82 (2.26 to 5.75) *
			NA	NA	2015–2021	-8.05 (-11.44 to -5.52) *
Hospitalization	$6,951.01	$5,602.51	-1.99 (-3.25 to -0.8) *	-8.24 (-12.52 to -5.06) *	2006–2009	6.95 (0.47 to 19.87) *
	NA	NA	NA	NA	2009–2016	-1.04 (-12.21 to 1.9)
	NA	NA	NA	NA	2016–2021	-8.24 (-16.73 to -1.4) *
Post-discharge	$421.97	$483.52	0.84 (0.12 to 1.49) *	0.91 (-2.04 to 3.43)	2006–2015	4.09 (3.13to 5.36) *
	NA	NA	NA	NA	2015–2018	-12.73 (-15.47 to -7.75) *
					2018–2021	5.92 (1.13to 15.72) *
Outpatient	$333.81	$623.71	4.31 (1.03 to 7.63) *	-1.69( -13.18 to 2.76)	2006–2014	9.85 (6.09 to 26.83) *
	NA	NA	NA	NA	2014–2021	-1.69 (-17.45 to 2.76)
ED-visit	$335.27	$993.55	7.21(6.44 to 8.12) *	3.9315 (0.94 to 6) *	2006–2008	1.06(-4.28 to 9.6)
	NA	NA	NA	NA	2008–2015	11.97 (9.93 to 17.36) *
	NA	NA	NA	NA	2015–2021	3.93 (0.58 to 6.19) *
Medicine	$548.64	$311.66	-3.1 (-6.86 to 0.69)	-7.17 (-20.17 -1.33) *	2006–2013	1.76 ( -4.87 to 36.9)
	NA	NA	NA	NA	2013–2021	-7.1657 (-29.59 to -1.22) *
Surgery	$1,139.40	$4,134.98	9.36 (8.61 to 10.19) *	2.89 (0.12 to 5.25) *	2006–2011	21.41(19.29 to 27.87) *
	NA	NA	NA	NA	2011–2015	13.69(9.62 to 17.01) *
	NA	NA	NA	NA	2015–2018	-11.96 (-14.93 to -7.25) *
	NA	NA	NA	NA	2018–2021	8.38(4.02 to 17.66) *
Hospitalization Rate	0.34%	0.31%	-0.66(-1.23 to 0.03)*	-2.66(-4.74 to -0.85)*	2006–2008	-6.02(-8.88 to -0.29)*
	NA	NA	NA	NA	2008–2018	1.49(0.98 to 4.4) *
	NA	NA	NA	NA	2018–2021	-4(-9.29 to -0.86) *
Sub phenotypes						
Congestive HF	$9,826.46	$7,902.51	-0.96(-2.01 to -0.11) *	-5.33(-9.23 to -2.26) *	2006–2015	4.24(0.51 to 7.13) *
	NA	NA	NA	NA	2015–2018	-13.87(-17.38 to 7.22)
	NA	NA	NA	NA	2018–2021	-2.3 (-9.01 to 7.26)
Systolic HF	$2,268.14	$6,885.87	7.3(6.15 to 8.55) *	-9.23(-11.14 to -7.58) *	2006–2008	104.61(83.74 to 126.69) *
	NA	NA	NA	NA	2008–2012	13.22(6.64 to 21.68) *
	NA	NA	NA	NA	2012–2021	-9.23 (-11.14 to -7.58) *
Diastolic HF	$6,391.69	$6,106.75	-0.43(-1.48 to 0.88)	-2.68(-7.34 to 1.39)	2006–2009	21.56(13.9 to 37.1) *
	NA	NA	NA	NA	2009–2014	1.69 (-2.65 to 6.65)
	NA	NA	NA	NA	2014–2017	-18.83 (-22.62 to -12.52) *
	NA	NA	NA	NA	2017–2021	-2.68 (-7.13 to 9.29)
Combined systolic and diastolic HF	$1,858.93	$7,283.21	8.6(7.34 to 10) *	-3.1(-6.95 to 1.32)	2006–2009	72.84(61.83 to 84.98) *
	NA	NA	NA	NA	2009–2014	2.35(-1.17 to 9.18)
	NA	NA	NA	NA	2014–2018	-12.3(-18.26 to -8.09) *
	NA	NA	NA	NA	2018–2021	0.17(-5.86 to 11.84)
Age Groups						
>=85	$9,563.96	$4,862.63	-3.66(-5.43to-1.99) *	-6.73(-13.65 to -0.57) *	2006–2015	3.38(0.28 to 7.27) *
	NA	NA	NA	NA	2015–2018	-25.14(-30.5 to -1.17) *
	NA	NA	NA	NA	2018–2021	0.36(-12.35 to19.96)
80–84	$6,428.62	$5,958.81	-3.32 (-8.74 to 2.23) ^b^	-3.32 (-8.74 to 2.23) ^b^	2016–2021	-3.32(-8.74 to 2.23) ^b^
75–79	$9,596.92	$6,819.84	-4.83(-9.5 to-0.62) *	-9.89(-19.12 to -3.38) *	2011–2014	8.11(-7.13 to 40.94)
	NA	NA	NA	NA	2014–2021	-9.89(-27.86 to -2.97) *
70–74	$7,859.35	$7,488.68	-1.19(-4.81 to 2.49)	-8.01(-20.4 to -3.11) *	2006–2014	5.20(0.92 to 23.09) *
	NA	NA	NA	NA	2014–2021	-8.01(-24.65 to -3.11) *
65–69	$11,166.02	$8,774.75	-0.85(-2.26 to0.45)	-7.27(-12.83 to-3.76) *	2006–2015	3.67(1.54 to 7.25) *
	NA	NA	NA	NA	2015–2021	-7.27(-13.49 to -3.76) *
60–64	$10,116.43	$15,860.57	3.67(2.22 to 5.26) *	-2.24(-7.23 to 3.24)	2006–2018	5.9(4.71 to 10.21) *
	NA	NA	NA	NA	2018–2021	-4.81(-16.91 to 3.23)
55–59	$8,935.64	$16,775.21	4.15(2.88 to 5.86) *	-1.09(-5.93 to 4.32)	2006–2019	6.12(5.05 to 10.86) *
	NA	NA	NA	NA	2019–2021	-7.82(-16.55 to 4.32)
50–54	$9,332.67	$18,033.51	6 (4.38 to7.59) ^b^*	6 (4.38 to7.59) ^b^*	2006–2021	6 (4.38 to7.59) ^b^*
< 50	$8,798.59	$21,390.47	7.35(5.07 to 9.61) ^b^*	7.35(5.07 to 9.61) ^b^*	2006–2021	7.35(5.07 to 9.61) ^b^*
Gender						
Female	$9,132.84	$7,498.38	-0.81(-2.09 to 0.15)	-4.14(-8.87 to -0.57) *	2006–2015	3.88(-1.04 to 7.19)
	NA	NA	NA	NA	2015–2018	-13.75(-17.85 to 8.01)
	NA	NA	NA	NA	2018–2021	-0.70(-8.71 to 10.45)
Male	$10,202.28	$8,916.62	-0.67(-1.45 to 0.07)	-7.81(-10.74 to -5.51) *	2006–2015	4.4(2.97 to 6.06) *
	NA	NA	NA	NA	2015–2021	-7.81(-10.74 to -5.51) *
Regions						
NorthEast	$8,638.66	$9,477.77	0.32(-0.71 to 1.88)	-5.92(-9.64 to -2.76) *	2006–2008	-4.19(-11.96 to 8.57)
	NA	NA	NA	NA	2008–2014	9.81(-10.5 to 18.76)
	NA	NA	NA	NA	2014–2021	-5.92(-10.3 to 0.81)
Mid-West	$8,992.51	$9,433.52	-0.22( -0.79 to 0.33)	-3.58(-5.05 to -2.48) *	2006–2013	3.76(2.35 to 5.72) *
	NA	NA	NA	NA	2013–2021	-3.58(-5.05 to -2.48) *
South	$9,372.37	$6,646.12	-1.7(-2.9 to -0.44) *	-9.42(-13.88 to -5.14) *	2006–2015	6.94(3.67 to 10.55) *
	NA	NA	NA	NA	2015–2018	-20.74(-24.68 to 9.72)
	NA	NA	NA	NA	2018–2021	-5.3(-13.67 to 7.12)
West	$12,255.48	$7,377.87	-2.36(-4.65 to 0.29)	-7.32(-15.39 to -2.17) *	2006–2016	0.22(-2 to 20.08)
	NA	NA	NA	NA	2016–2021	-7.32(-24.45 to -2.17) *
Payment by insurance payers						
Commercial	$8,865.32	$19,273.59	6(3.6 to 8.9) *	-0.34(-8.09 to 6.12)	2006–2017	8.41(6.73 to 27.43) *
	NA	NA	NA	NA	2017–2021	-0.34(-19.22 to 6.12)
Managed Medicaid	$8,112.72	$7,303.25	1.46(-0.5 to 3.41) ^b^	1.46(-0.5 to 3.41) ^b^	2006–2021	1.46(-0.5 to 3.41) ^b^
Medicare Risk	$7,255.85	$10,833.8	2.62(1.44 to 3.82) *	-1.15(-4.96 to 2.31)	2006–2017	4.03(3.25 to 8.5) *
	NA	NA	NA	NA	2017–2021	-1.14(-10.89 to 2.31)
Self-Insured	$6,587.78	$10,490.95	2.75(-1.34 to 6.4)	-1.97(-10.48 to 1.33)	2006–2009	16.5(3.22 to 48.27) *
	NA	NA	NA	NA	2009–2017	-1.97(-17.57 to 1.33)
Medicare Cost	$11,385.73	$1,748.98	-10.36(-14.37 to -6.86) *	-17.8(-31.07 to -5.32) *	2006–2015	3.85(-12.98 to 15.45)
	NA	NA	NA	NA	2015–2018	-45.03(-53.6 to 19.31)
	NA	NA	NA	NA	2018–2021	-6(-30.64 to 38.4)
Self-payment						
Commercial	$3,522.21	$3,336.48	0.29(-1.11 to 1.71)	2.69(-3.27 to 9.3)	2006–2013	4.56(1.65 to 10.26) *
	NA	NA	NA	NA	2013–2016	-12.55(-17.25 to -2.68) *
	NA	NA	NA	NA	2016–2021	2.69(-3.26 to 17.01)
Managed Medicaid	$477.08	$2,227.43	14.13(6.49 to21.49) *	4.82(-16.04 to 11.08)	2006–2011	35.29(15.3 to 132.88) *
	NA	NA	NA	NA	2011–2021	4.82(-27.98 to 11.08)
Medicare Risk	$441.20	$1,038.64	6.12(2.27 to 10.04) ^b^*	6.12(2.27 to 10.04) ^b^*	2006–2021	6.12(2.27 to 10.04) ^b^ *
Self-Insured	$1,177.53	$503.10	-13.2(-25.69 to 6.01)	6.17(-38.45 to 64.77)	2006–2010	51.75(2.97 to 360.86) *
	NA	NA	NA	NA	2010–2013	-68.5(-81.48 to -38.76) *
	NA	NA	NA	NA	2013–2021	6.17(-37.63 to 226.93)
Medicare Cost	$10,589.26	$488.61	-19.2(-24.32 to 14.18) *	-42.18(-56.31 to -31.25) *	2006–2015	0.99(-8.29 to 17.17)
	NA	NA	NA	NA	2015–2021	-42.18(-57.17 to -31.25) *

Abbreviations: AAPC: average annual percent change; APC: annual percent change; NA: not applicable

^a^ Joinpoint allowed up to 3 joinpoints

^b^ No joinpoint was detected for these strata and outcomes; APC was constant and equal to AAPC.

* Indicates that the AAPC significantly differs from zero at the alpha = 0.05 level

The hospitalization rate (shown in supplemental material eTable 3) decreased from 34% in 2006 to 31% in 2021, with an AAPC of -0.66% (95% CI: −1.23% to − 0.03%) (Table 2). Significant decreases happened in 2006–2008 and 2018–2021 with APCs of -6.02% (95% CI: -8.88% to -0.29%) and − 4% (95% CI: -9.29% to -0.86%) (Table 2). From 2009 to 2017 hospitalization rate increased from 30 to 35% with an APC of 1.49% (95 CI: 0.98–4.4%).

### Sub-phenotypes, age group, gender, comorbidities and geographic

**Fig. 2:**
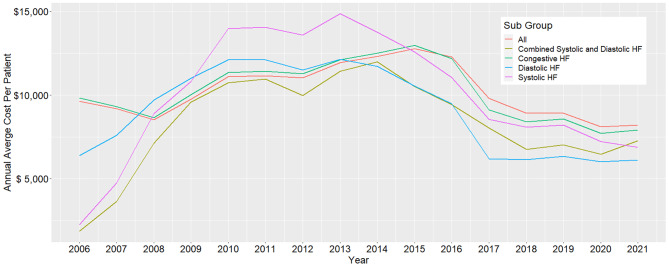
Annual trends in average cost per patient and subphenotype from 2006 to 2021. The subgroups include all HF cases, congestive HF (under ICD10 code I50), systolic HF (under ICD10 code I502), diastolic HF (under ICD10 code I503 code) and combined systolic and diastolic HF (under ICD10 code I504)

The annual trend analysis for HF sub-phenotypes is presented in Table 2; Fig. 2. The data indicate fluctuations in the average costs for all HF subgroups over the years. Specifically, the overall trend observed for congestive HF was an increase in payments from $9,826.46 in 2006 to $12,970.73 in 2015 with an APC of 4.24% (95% CI:0.51–7.13%). This was followed by drops in payments in 2016–2018 with APCs of -13.87% (95% CI: -17.38–7.22%) and again from 2019 to 2021 with APCs of -2.3% (95% CI: -9.01–7.26%). This represented an overall 19.58% drop in 2021 compared to 2006. The annual cost breakdown by sub-phenotypes is presented in the supplemental material eTable 4.

The analysis of annual average medical costs associated with age groups from 2006 to 2021 reveals that the medical costs per patient tend to increase as the age group decreases. Comparing subjects in the each of the age groups > = 85, 80–84, 75–79, 70–74, and 65–69 presented an increasing trend with negative AAPCs of -3.66% (95% CI: -5.43% to-1.99%), -3.32% (95% CI:-8.74–2.23%), -4.83% (95% CI:-9.5% to -0.62%), -1.19% (95% CI:-4.81–2.49%) and − 0.85% (95% CI:-2.26–0.45%), respectively. The age groups 60–64, 55–59, 50–54, and < 50 exhibited a continued annual increasing trend with positive AAPCs of 3.67% (95% CI: 2.22–5.26%), 4.15% (95% CI: 2.88–5.86%), 6% (95% CI:4.38–7.59%) and 7.35% (95% CI:5.07–9.61%) (shown in Table 2 and supplemental material Fig. 2). The annual cost breakdown by age group is presented in the supplemental material eTable 5.

Males consistently had higher costs than females over the entire period (shown in supplemental material eFigure 3). The annual average medical costs of each comorbidity index group from 2006 to 2021 are shown in supplemental material eTable 6 and eFigure 4. The data reveals a general trend where the average cost increases as the comorbidity index increases.

This study investigated the mean medical expenses per patient across the United States, categorized into four census regions: Northeast, Midwest, South, and West. The annual average cost per patient trends are presented in supplemental material eFigure 5 and Table 2. In addition, supplemental material eTable 8 shows that the average cost per patient over 16 years across 50 US states varies widely. The highest average cost per patient was in Kansas ($15,624.27), followed by Vermont ($14,888.58) and Minnesota ($14,696.76), while the lowest cost is in Delaware ($7,622.25), New Hampshire ($7,482.90) and Idaho ($6,664.28).

### Insurance payment and self-payment

**Fig. 3:**
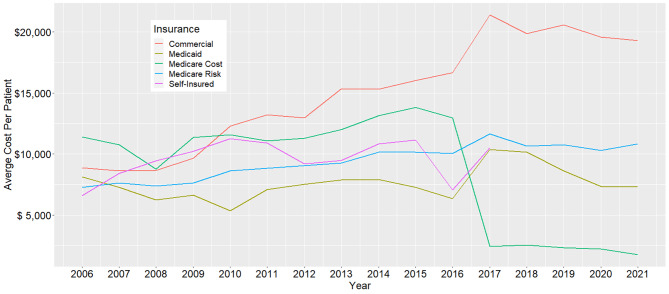
Annual trends in average cost per patient and insurance payment type from 2006 to 2021

The study considered five types of insurance payments, namely Commercial, Managed Medicaid, Medicare Risk, Self-Insured, and Medicare Cost. Insurance payment and self-payment analyses were also based on these five categories. The study analyzed the trends of the annual average cost per patient, as illustrated in Fig. 3; Table 2. Commercial insurance payment presented an increasing stage of 2006–2017 with an APC of 8.41% (95%CI: 6.73–27.43%), and a decreasing period of 2017–2021 with an APC of -0.34% (95%CI: -19.22–6.12%). Managed Medicaid insurance payment decreased with an AAPC of -1.46% (95%CI: -0.5–3.41%). Medicare Risk insurance payment increased with an AAPC of 2.62% (95%CI: 1.44–3.82%). Self-insured insurance payment increased with an AAPC of 2.75% (95%CI: -1.34–6.4%). Medicare Cost insurance payment decreased with an AAPC of -10.36%(95%CI: -14.37% to -6.86%), presenting one increasing period of 2006–2015 with an APC of 3.85%(95%CI: -12.98–15.45%) and two decreasing stages 2015–2018 and 2018–2021 with APCs of -45.03%(95%CI: -53.6–19.31%) and − 6%(95%CI: -30.64–38.4%). In addition, the study analyzed the average self-payment, and results are presented in supplemental material eFigure 6 and Table 2.

## Discussion

This cross-sectional study describes national trends in HF expenditure among commercially insured patients by characteristics including age, gender, comorbidity, payment types, and region. The average total annual HF cost per patient has decreased by -8.05% (95%CI: -11.44% to -5.52%) since 2015. This trend was consistent across various subtypes of HF. The average total annual cost per patient increased from 2006 to 2015, with a subsequent decrease until 2021. The subgroup analysis indicates that the average cost per patient under the group of Medicare Cost insurance declined after 2016 (shown in Fig. 3), the left groups showing an increasing trend. In addition, the age group analysis indicated that only patients above 65 presented a decreasing trend after 2016 (shown in supplemental material eFigure 1). The cause of the average cost decline after 2016 is the Medicare Cost insurance payment that showed a significant decrease after 2016, which accompanied changes in Medicare Cost insurance plans following the implementation of the Medicare Access and CHIP Reauthorization Act (MACRA) in 2015 [12]. MACRA aimed to cut Medicare costs by changing physician reimbursement models to promote value over volume, including transitioning away from Medicare Cost plans in certain areas. The bimodal trends in cost could be attributed, in part, to the Hospital Readmission Reduction Program for HF patients implemented by the Centers for Medicare & Medicaid Services (CMS). This initiative prompted hospitals to take aggressive measures in optimizing HF care to evade financial penalties, potentially influencing the declining costs observed in the latter half of the study.

The hospitalization rate analysis revealed decreased hospitalization rates from 2006 to 2021. However, there were significant fluctuations during different periods, with notable decreases in 2006–2008 and 2018–2021 and an increase from 2009 to 2017. For the majority of patients with HF, the disease follows a pattern of alternating periods of relative stability, which can vary in duration, and episodes of deteriorating symptoms that frequently necessitate hospitalization [13]. It is important to note that each subsequent hospitalization heightens the mortality risk and exacerbates the decline in quality of life [14–16]. Moreover, it is noteworthy that average annual hospitalization and medication costs per patient decreased, while outpatient, post-discharge, ED visits, and surgery costs increased over the study period. These findings reflect the complex nature of HF management, shifting towards more outpatient care and interventions, such as intracardiac monitoring systems [17]. These findings are consistent with previous studies that have identified a similar pattern of hospitalizations for HF [18, 19].

The analysis of age groups demonstrated that younger patients showed increasing trends in medical expenses, possibly due to the higher prevalence of risk factors and comorbidities in the age groups [20–23]. Older patients are more likely to have risk factors and co-morbidities than younger patients. All comorbidity classes showed declining costs from 2006 to 2021. Younger patients, however, particularly below the age of 65 who did not qualify for Medicare, had rising costs from 2006 to 2021. It appears MACRA disproportionately benefited Medicare patients at the expense of younger, non-Medicare patients. Gender variation analysis revealed that males consistently had higher medical costs than females over the entire study period. This finding may be attributed to differences in disease severity, treatment patterns, and healthcare-seeking behaviors between genders [24]. The analysis of comorbidities demonstrated that patients with higher comorbidity burdens tended to have higher medical costs associated with their HF treatment [25]. This finding highlights the importance of comprehensive management of comorbid conditions in HF patients to reduce the economic burden and improve patient outcomes.

The analysis of geographic differences indicated variations in average costs per patient across different regions and states in the United States. The northeast and midwest regions showed relatively stable trends, with slight increases or decreases over time. In contrast, the south and west regions exhibited more significant fluctuations in average costs. These findings suggest that targeted interventions and resource allocation strategies may be required to address the regional and state-level differences in HF-related costs, as indicated by prior studies [26, 27].

Moreover, we recognize the significant drop in ED visits, outpatient visits, and hospitalizations observed during the pandemic, as documented in the literature [28]. However, due to the limitations of claims data, including potential delays in data reporting and processing, the exact impact on our study variables may not be fully captured. We acknowledge this limitation and emphasize the need for a cautious interpretation of the findings, particularly during the pandemic period.

In conclusion, this study provides valuable insights into the annual cost trends and differences per sub-phenotype, age group, gender, comorbidity, and geographic region for commercially insured HF patients. Moreover, the analysis of insurance payment patterns underscores the importance of understanding reimbursement mechanisms and their implications for access to care and financial support for HF patients.

### Limitation

The analysis is based on administrative claims data, which may not capture all relevant clinical information. Moreover, the study is observational in nature, and causality cannot be established between variables.

### Electronic supplementary material

Below is the link to the electronic supplementary material.

Supplementary Material 1

## Data Availability

No datasets were generated or analysed during the current study.
