# Supplementary material for: Trends in heart failure costs for commercially insured patients in the United States (2006–2021)
Source: BMC Health Serv Res. 2024 Jul 8;24:780. doi: 10.1186/s12913-024-11240-4 (PMC11232302; doi:10.1186/s12913-024-11240-4)

**Trends in Heart Failure Cost for Commercial Insured Patients in the USA (2006-2021)**

**Supplementary Online Content**

**eTable 1. The ICD-9/10 code list in the study.**

**eTable 2. Annual average costs per patient, broken down by cost category 2006-2021.**

**eTable 3. Hospitalization rate, mean stay with standard deviation (SD), and median stay for each year from 2006 to 2021.**

**eTable 4. Annual average costs per patient and sub-phenotype 2006-2021.**

**eTable 5. Annual average costs per patient and age group 2006-2021.**

**eTable 6. The annual average costs per patient and comorbidity index group from 2006 to 2021.**

**eTable 7.** **Annual average costs per patient and region 2006-2021.**

**eTable 8.** **The average costs per patient and state over 2006-2021.**

**eTable 9. Annual average costs per patient and insurance type 2006-2021.**

**eTable 10. Annual average self-payment per patient and payment type 2006-2021.**

**eFigure 1. PharMetrics Plus for Academics Enrollee Characteristics.**

**eFigure 2. The annual average costs per patient and age group trends from 2006 to 2021.**

**eFigure 3. The annual average costs per patient and gender trends from 2006 to 2021.**

**eFigure 4. The annual average cost per patient and comorbidity index group trends from 2006 to 2021**.

**eFigure 5. The annual average medical costs per patient and region trends from 2006-2021.**

**eFigure 6.** **The annual average self-payment per patient and insurance type trends 2006-2021.**

**eTable 1. The ICD-9/10 code list in the study.**

| **ICD 10 CM codes** | **Description** |
| --- | --- |
| I0981 | rheumatic heart failure |
| I110 | hypertensive heart disease with heart failure |
| I130 | hypertensive heart and chronic kidney disease with heart failure and stage 1 through stage 4 chronic kidney disease, or unspecified chronic kidney disease |
| I132 | hypertensive heart and chronic kidney disease with heart failure and with stage 5 chronic kidney disease, or end stage renal disease |
| I50 | heart failure |
| I501 | left ventricular failure, unspecified |
| I502 | systolic (congestive) heart failure |
| I5020 | unspecified systolic (congestive) heart failure |
| I5021 | acute systolic (congestive) heart failure |
| I5022 | chronic systolic (congestive) heart failure |
| I5023 | acute on chronic systolic (congestive) heart failure |
| I503 | diastolic (congestive) heart failure |
| I5030 | unspecified diastolic (congestive) heart failure |
| I5031 | acute diastolic (congestive) heart failure |
| I5032 | chronic diastolic (congestive) heart failure |
| I5033 | acute on chronic diastolic (congestive) heart failure |
| I504 | combined systolic (congestive) and diastolic (congestive) heart failure |
| I5040 | unspecified combined systolic (congestive) and diastolic (congestive) heart failure |
| I5041 | acute combined systolic (congestive) and diastolic (congestive) heart failure |
| I5042 | chronic combined systolic (congestive) and diastolic (congestive) heart failure |
| I5043 | acute on chronic combined systolic (congestive) and diastolic (congestive) heart failure |
| I508 | other heart failure |
| I50810 | right heart failure, unspecified |
| I50811 | acute right heart failure |
| I50812 | chronic right heart failure |
| I50813 | acute on chronic right heart failure |
| I50814 | right heart failure due to left heart failure |
| I5082 | biventricular heart failure |
| I5083 | high output heart failure |
| I5084 | end stage heart failure |
| I5089 | other heart failure |
| I509 | heart failure, unspecified |
| I9713 | postprocedural heart failure |
| I97130 | postprocedural heart failure following cardiac surgery |
| I97131 | postprocedural heart failure following other surgery |
| **ICD 9 CM codes** | **Description** |
| 428 | heart failure |
| 4280 | congestive heart failure, unspecified |
| 4281 | left heart failure |
| 4282 | systolic heart failure |
| 4283 | diastolic heart failure |
| 4284 | combined systolic and diastolic heart failure |
| 4289 | heart failure, unspecified |
| 39891 | rheumatic heart failure (congestive) |
| 40201 | malignant hypertensive heart disease with congestive heart failure |
| 40211 | benign hypertensive heart disease with congestive heart failure |
| 40291 | unspecified hypertensive heart disease with congestive heart failure |
| 40401 | hypertensive heart and chronic kidney disease, malignant, with heart failure and with chronic kidney disease stage I through stage IV, or unspecified |
| 40403 | hypertensive heart and chronic kidney disease, malignant, with heart failure and with chronic kidney disease stage V or end stage renal disease |
| 40411 | hypertensive heart and chronic kidney disease, benign, with heart failure and with chronic kidney disease stage I through stage IV, or unspecified |
| 40413 | hypertensive heart and chronic kidney disease, benign, with heart failure and chronic kidney disease stage V or end stage renal disease |
| 40491 | hypertensive heart and chronic kidney disease, unspecified, with heart failure and with chronic kidney disease stage I through stage IV, or unspecified |
| 40493 | hypertensive heart and chronic kidney disease, unspecified, with heart failure and chronic kidney disease stage V or end stage renal disease |
| 42820 | unspecified systolic heart failure |
| 42821 | acute systolic heart failure |
| 42822 | chronic systolic heart failure |
| 42823 | acute on chronic systolic heart failure |
| 42830 | unspecified diastolic heart failure |
| 42831 | acute diastolic heart failure |
| 42832 | chronic diastolic heart failure |
| 42833 | acute on chronic diastolic heart failure |
| 42840 | unspecified combined systolic and diastolic heart failure |
| 42841 | acute combined systolic and diastolic heart failure |
| 42842 | chronic combined systolic and diastolic heart failure |
| 42843 | acute on chronic combined systolic and diastolic heart failure |

**eTable 2.** **Annual average costs per patient, broken down by cost category 2006-2021.** This table provides information on the average total medical costs and costs broken down by categories, including hospitalization, post-discharge care, outpatient care, medicine, and surgery, related to heart failure treatment per patient.

| **Year** | **Total** | **Hospitalization** | **Post-Discharge** | **Outpatient** | **ED-Visit** | **Medicine** | **Surgery** |
| --- | --- | --- | --- | --- | --- | --- | --- |
| 2006 | $9,636.99 | $6,951.01 | $421.97 | $333.81 | $335.27 | $548.64 | $1,139.40 |
| 2007 | $9,195.69 | $7,546.56 | $456.28 | $355.68 | $355.40 | $515.12 | $1,235.09 |
| 2008 | $8,512.91 | $7,863.59 | $464.13 | $334.04 | $337.54 | $489.35 | $1,528.95 |
| 2009 | $9,759.04 | $8,703.28 | $506.79 | $376.33 | $413.00 | $411.28 | $1,980.87 |
| 2010 | $11,106.83 | $8,910.69 | $511.21 | $417.51 | $438.09 | $432.65 | $2,364.67 |
| 2011 | $11,149.38 | $8,644.14 | $518.30 | $435.63 | $467.10 | $473.08 | $2,868.01 |
| 2012 | $11,027.07 | $7,268.49 | $542.48 | $495.09 | $523.76 | $638.79 | $3,168.20 |
| 2013 | $11,926.43 | $7,888.06 | $560.76 | $625.30 | $620.80 | $600.77 | $3,714.23 |
| 2014 | $12,312.13 | $7,932.53 | $613.43 | $681.83 | $691.91 | $544.49 | $4,215.52 |
| 2015 | $12,763.90 | $8,563.68 | $604.86 | $689.47 | $766.73 | $555.78 | $4,605.37 |
| 2016 | $12,285.50 | $8,620.31 | $583.69 | $695.20 | $877.99 | $347.86 | $4,494.56 |
| 2017 | $9,796.24 | $7,135.60 | $425.71 | $519.49 | $766.44 | $380.09 | $3,407.82 |
| 2018 | $8,929.39 | $6,399.32 | $420.44 | $548.05 | $848.11 | $276.64 | $3,345.62 |
| 2019 | $8,923.22 | $5,839.49 | $451.79 | $593.43 | $898.64 | $357.72 | $3,485.21 |
| 2020 | $8,098.21 | $5,392.60 | $462.91 | $550.96 | $919.79 | $383.59 | $3,805.67 |
| 2021 | $8,201.89 | $5,602.51 | $483.52 | $623.71 | $993.55 | $311.66 | $4,134.98 |

**eTable 3. Hospitalization rate, mean stay with standard deviation (SD), and median stay for each year from 2006 to 2021.**

| **Year** | **Hospitalization Rate (%)** | **Mean stay ± SD** | **Median stay** |
| --- | --- | --- | --- |
| 2006 | 0.34 | 7±10 | 4 |
| 2007 | 0.32 | 7±12 | 4 |
| 2008 | 0.29 | 7±12 | 4 |
| 2009 | 0.30 | 7±12 | 4 |
| 2010 | 0.31 | 7±11 | 4 |
| 2011 | 0.31 | 7±11 | 4 |
| 2012 | 0.33 | 7±10 | 4 |
| 2013 | 0.33 | 7±11 | 4 |
| 2014 | 0.33 | 7±12 | 4 |
| 2015 | 0.32 | 7±15 | 4 |
| 2016 | 0.32 | 7±16 | 4 |
| 2017 | 0.35 | 7±13 | 4 |
| 2018 | 0.34 | 7±10 | 4 |
| 2019 | 0.34 | 7±10 | 4 |
| 2020 | 0.31 | 7±10 | 5 |
| 2021 | 0.31 | 8±10 | 5 |

**eTable 4. Annual average costs per patient and sub-phenotype 2006-2021.** The subgroups include all heart failure cases, congestive heart failure (under ICD10 code I50), systolic heart failure (under ICD10 code I502), diastolic heart failure (under ICD10 code I503 code), and combined systolic and diastolic heart failure (under ICD10 code I504).

| Year | Subgroup | Average Cost |
| --- | --- | --- |
| 2006 | All | $9,636.99 |
| 2007 | All | $9,195.69 |
| 2008 | All | $8,512.91 |
| 2009 | All | $9,759.04 |
| 2010 | All | $11,106.83 |
| 2011 | All | $11,149.38 |
| 2012 | All | $11,027.07 |
| 2013 | All | $11,926.43 |
| 2014 | All | $12,312.13 |
| 2015 | All | $12,763.90 |
| 2016 | All | $12,285.50 |
| 2017 | All | $9,796.24 |
| 2018 | All | $8,929.39 |
| 2019 | All | $8,923.22 |
| 2020 | All | $8,098.21 |
| 2021 | All | $8,201.89 |
| 2006 | Congestive HF | $9,826.46 |
| 2007 | Congestive HF | $9,312.75 |
| 2008 | Congestive HF | $8,649.11 |
| 2009 | Congestive HF | $10,014.01 |
| 2010 | Congestive HF | $11,358.40 |
| 2011 | Congestive HF | $11,423.40 |
| 2012 | Congestive HF | $11,270.76 |
| 2013 | Congestive HF | $12,115.87 |
| 2014 | Congestive HF | $12,494.93 |
| 2015 | Congestive HF | $12,970.73 |
| 2016 | Congestive HF | $12,195.56 |
| 2017 | Congestive HF | $9,105.74 |
| 2018 | Congestive HF | $8,408.40 |
| 2019 | Congestive HF | $8,562.14 |
| 2020 | Congestive HF | $7,706.98 |
| 2021 | Congestive HF | $7,902.51 |
| 2006 | Systolic HF | $2,268.14 |
| 2007 | Systolic HF | $4,742.07 |
| 2008 | Systolic HF | $8,880.82 |
| 2009 | Systolic HF | $10,806.86 |
| 2010 | Systolic HF | $13,971.47 |
| 2011 | Systolic HF | $14,053.98 |
| 2012 | Systolic HF | $13,591.62 |
| 2013 | Systolic HF | $14,854.88 |
| 2014 | Systolic HF | $13,752.14 |
| 2015 | Systolic HF | $12,568.79 |
| 2016 | Systolic HF | $11,045.00 |
| 2017 | Systolic HF | $8,560.43 |
| 2018 | Systolic HF | $8,081.39 |
| 2019 | Systolic HF | $8,214.93 |
| 2020 | Systolic HF | $7,230.97 |
| 2021 | Systolic HF | $6,885.87 |
| 2006 | Diastolic HF | $6,391.69 |
| 2007 | Diastolic HF | $7,588.63 |
| 2008 | Diastolic HF | $9,702.54 |
| 2009 | Diastolic HF | $11,002.82 |
| 2010 | Diastolic HF | $12,103.05 |
| 2011 | Diastolic HF | $12,102.54 |
| 2012 | Diastolic HF | $11,503.54 |
| 2013 | Diastolic HF | $12,145.62 |
| 2014 | Diastolic HF | $11,710.92 |
| 2015 | Diastolic HF | $10,536.11 |
| 2016 | Diastolic HF | $9,480.19 |
| 2017 | Diastolic HF | $6,198.74 |
| 2018 | Diastolic HF | $6,148.85 |
| 2019 | Diastolic HF | $6,340.41 |
| 2020 | Diastolic HF | $6,028.66 |
| 2021 | Diastolic HF | $6,106.75 |
| 2006 | Combined Systolic and Diastolic HF | $1,858.93 |
| 2007 | Combined Systolic and Diastolic HF | $3,636.41 |
| 2008 | Combined Systolic and Diastolic HF | $7,115.93 |
| 2009 | Combined Systolic and Diastolic HF | $9,576.23 |
| 2010 | Combined Systolic and Diastolic HF | $10,728.17 |
| 2011 | Combined Systolic and Diastolic HF | $10,959.70 |
| 2012 | Combined Systolic and Diastolic HF | $9,982.21 |
| 2013 | Combined Systolic and Diastolic HF | $11,411.40 |
| 2014 | Combined Systolic and Diastolic HF | $11,975.78 |
| 2015 | Combined Systolic and Diastolic HF | $10,503.17 |
| 2016 | Combined Systolic and Diastolic HF | $9,442.02 |
| 2017 | Combined Systolic and Diastolic HF | $8,022.26 |
| 2018 | Combined Systolic and Diastolic HF | $6,753.98 |
| 2019 | Combined Systolic and Diastolic HF | $7,033.49 |
| 2020 | Combined Systolic and Diastolic HF | $6,455.01 |
| 2021 | Combined Systolic and Diastolic HF | $7,283.21 |

**eTable 5. Annual average costs per patient and age group 2006-2021.** Age Group 1: >=85 years, Age Group 2: 80-84 years, Age Group 3: 75-79 years, Age Group 4: 70-74 years, Age Group 5: 65-69 years, Age Group 6: 60-64 years, Age Group 7: 55-59 years, Age Group 8: 50-54 years, and Age Group 9: <50 years.

| Year | Age Group | Comorbid HF |
| --- | --- | --- |
| 2006 | Age Group =1 | $9,563.96 |
| 2007 | Age Group =1 | $9,143.51 |
| 2008 | Age Group =1 | $8,432.88 |
| 2009 | Age Group =1 | $9,469.59 |
| 2010 | Age Group =1 | $10,690.16 |
| 2011 | Age Group =1 | $10,407.92 |
| 2012 | Age Group =1 | $10,425.55 |
| 2013 | Age Group =1 | $10,783.28 |
| 2014 | Age Group =1 | $11,302.03 |
| 2015 | Age Group =1 | $11,702.49 |
| 2016 | Age Group =1 | $11,260.19 |
| 2017 | Age Group =1 | $5,222.81 |
| 2018 | Age Group =1 | $5,084.92 |
| 2019 | Age Group =1 | $5,467.31 |
| 2020 | Age Group =1 | $5,056.24 |
| 2021 | Age Group =1 | $4,862.63 |
| 2016 | Age Group =2 | $6,428.62 |
| 2017 | Age Group =2 | $7,931.16 |
| 2018 | Age Group =2 | $6,105.22 |
| 2019 | Age Group =2 | $6,150.96 |
| 2020 | Age Group =2 | $6,055.37 |
| 2021 | Age Group =2 | $5,958.81 |
| 2011 | Age Group =3 | $9,596.92 |
| 2012 | Age Group =3 | $9,315.64 |
| 2013 | Age Group =3 | $11,384.55 |
| 2014 | Age Group =3 | $12,152.49 |
| 2015 | Age Group =3 | $11,620.05 |
| 2016 | Age Group =3 | $12,011.41 |
| 2017 | Age Group =3 | $5,990.00 |
| 2018 | Age Group =3 | $6,366.55 |
| 2019 | Age Group =3 | $6,902.68 |
| 2020 | Age Group =3 | $6,308.30 |
| 2021 | Age Group =3 | $6,819.84 |
| 2006 | Age Group =4 | $7,859.35 |
| 2007 | Age Group =4 | $9,280.55 |
| 2008 | Age Group =4 | $7,566.92 |
| 2009 | Age Group =4 | $9,386.96 |
| 2010 | Age Group =4 | $11,279.28 |
| 2011 | Age Group =4 | $10,954.87 |
| 2012 | Age Group =4 | $9,269.64 |
| 2013 | Age Group =4 | $11,690.96 |
| 2014 | Age Group =4 | $11,781.67 |
| 2015 | Age Group =4 | $12,389.32 |
| 2016 | Age Group =4 | $11,376.00 |
| 2017 | Age Group =4 | $8,337.81 |
| 2018 | Age Group =4 | $7,672.17 |
| 2019 | Age Group =4 | $7,493.78 |
| 2020 | Age Group =4 | $7,276.79 |
| 2021 | Age Group =4 | $7,488.68 |
| 2006 | Age Group =5 | $11,166.02 |
| 2007 | Age Group =5 | $9,399.39 |
| 2008 | Age Group =5 | $8,245.07 |
| 2009 | Age Group =5 | $9,961.86 |
| 2010 | Age Group =5 | $11,269.38 |
| 2011 | Age Group =5 | $11,802.48 |
| 2012 | Age Group =5 | $11,960.26 |
| 2013 | Age Group =5 | $12,032.27 |
| 2014 | Age Group =5 | $12,767.74 |
| 2015 | Age Group =5 | $13,316.71 |
| 2016 | Age Group =5 | $12,317.05 |
| 2017 | Age Group =5 | $10,521.53 |
| 2018 | Age Group =5 | $10,442.90 |
| 2019 | Age Group =5 | $9,533.14 |
| 2020 | Age Group =5 | $8,478.91 |
| 2021 | Age Group =5 | $8,774.75 |
| 2006 | Age Group =6 | $10,116.43 |
| 2007 | Age Group =6 | $10,407.92 |
| 2008 | Age Group =6 | $9,088.59 |
| 2009 | Age Group =6 | $11,482.29 |
| 2010 | Age Group =6 | $11,948.00 |
| 2011 | Age Group =6 | $13,462.81 |
| 2012 | Age Group =6 | $12,109.19 |
| 2013 | Age Group =6 | $15,371.38 |
| 2014 | Age Group =6 | $15,291.14 |
| 2015 | Age Group =6 | $14,771.61 |
| 2016 | Age Group =6 | $14,482.96 |
| 2017 | Age Group =6 | $19,465.35 |
| 2018 | Age Group =6 | $19,609.00 |
| 2019 | Age Group =6 | $18,034.56 |
| 2020 | Age Group =6 | $17,533.52 |
| 2021 | Age Group =6 | $15,860.57 |
| 2006 | Age Group =7 | $8,935.64 |
| 2007 | Age Group =7 | $9,110.83 |
| 2008 | Age Group =7 | $9,063.66 |
| 2009 | Age Group =7 | $9,920.46 |
| 2010 | Age Group =7 | $12,913.11 |
| 2011 | Age Group =7 | $12,091.67 |
| 2012 | Age Group =7 | $13,257.14 |
| 2013 | Age Group =7 | $13,214.87 |
| 2014 | Age Group =7 | $14,346.68 |
| 2015 | Age Group =7 | $14,366.02 |
| 2016 | Age Group =7 | $13,360.58 |
| 2017 | Age Group =7 | $19,041.74 |
| 2018 | Age Group =7 | $18,411.59 |
| 2019 | Age Group =7 | $19,265.43 |
| 2020 | Age Group =7 | $16,228.64 |
| 2021 | Age Group =7 | $16,775.21 |
| 2006 | Age Group =8 | $9,332.67 |
| 2007 | Age Group =8 | $8,560.27 |
| 2008 | Age Group =8 | $8,420.21 |
| 2009 | Age Group =8 | $9,873.90 |
| 2010 | Age Group =8 | $10,107.30 |
| 2011 | Age Group =8 | $12,065.58 |
| 2012 | Age Group =8 | $11,836.01 |
| 2013 | Age Group =8 | $12,431.74 |
| 2014 | Age Group =8 | $14,034.72 |
| 2015 | Age Group =8 | $14,175.05 |
| 2016 | Age Group =8 | $12,828.90 |
| 2017 | Age Group =8 | $21,064.05 |
| 2018 | Age Group =8 | $17,103.15 |
| 2019 | Age Group =8 | $16,445.23 |
| 2020 | Age Group =8 | $20,498.33 |
| 2021 | Age Group =8 | $18,033.51 |
| 2006 | Age Group =9 | $8,798.59 |
| 2007 | Age Group =9 | $8,193.17 |
| 2008 | Age Group =9 | $9,171.67 |
| 2009 | Age Group =9 | $9,830.41 |
| 2010 | Age Group =9 | $11,999.72 |
| 2011 | Age Group =9 | $12,441.28 |
| 2012 | Age Group =9 | $14,873.36 |
| 2013 | Age Group =9 | $14,841.21 |
| 2014 | Age Group =9 | $13,300.77 |
| 2015 | Age Group =9 | $14,700.96 |
| 2016 | Age Group =9 | $14,171.77 |
| 2017 | Age Group =9 | $25,914.75 |
| 2018 | Age Group =9 | $21,965.52 |
| 2019 | Age Group =9 | $24,754.41 |
| 2020 | Age Group =9 | $18,879.29 |
| 2021 | Age Group =9 | $21,390.47 |

**eTable 6. The annual average costs per patient and comorbidity index group from 2006 to 2021.**

| Comorbidity index group | year | comorbid HF |
| --- | --- | --- |
| 1 | 2006 | $2,115.31 |
| 1 | 2007 | $2,011.39 |
| 1 | 2008 | $1,446.87 |
| 1 | 2009 | $1,554.81 |
| 1 | 2010 | $1,603.99 |
| 1 | 2011 | $1,285.60 |
| 1 | 2012 | $1,207.60 |
| 1 | 2013 | $1,158.79 |
| 1 | 2014 | $1,404.79 |
| 1 | 2015 | $1,425.95 |
| 1 | 2016 | $1,688.57 |
| 1 | 2017 | $1,658.19 |
| 1 | 2018 | $1,535.72 |
| 1 | 2019 | $1,340.81 |
| 1 | 2020 | $967.48 |
| 1 | 2021 | $946.39 |
| 2 | 2006 | $5,216.92 |
| 2 | 2007 | $4,813.17 |
| 2 | 2008 | $3,940.93 |
| 2 | 2009 | $4,303.12 |
| 2 | 2010 | $4,781.47 |
| 2 | 2011 | $4,242.38 |
| 2 | 2012 | $3,851.84 |
| 2 | 2013 | $4,073.08 |
| 2 | 2014 | $4,297.83 |
| 2 | 2015 | $4,478.55 |
| 2 | 2016 | $4,810.91 |
| 2 | 2017 | $3,783.15 |
| 2 | 2018 | $3,550.25 |
| 2 | 2019 | $3,493.95 |
| 2 | 2020 | $2,417.05 |
| 2 | 2021 | $2,323.93 |
| 3 | 2006 | $11,923.17 |
| 3 | 2007 | $10,949.99 |
| 3 | 2008 | $9,885.69 |
| 3 | 2009 | $11,067.91 |
| 3 | 2010 | $12,041.02 |
| 3 | 2011 | $10,926.84 |
| 3 | 2012 | $10,241.44 |
| 3 | 2013 | $10,736.61 |
| 3 | 2014 | $10,152.76 |
| 3 | 2015 | $10,779.45 |
| 3 | 2016 | $11,542.66 |
| 3 | 2017 | $8,365.65 |
| 3 | 2018 | $8,963.01 |
| 3 | 2019 | $7,176.29 |
| 3 | 2020 | $7,622.50 |
| 3 | 2021 | $6,458.92 |
| 4 | 2006 | $20,847.23 |
| 4 | 2007 | $20,418.17 |
| 4 | 2008 | $17,808.80 |
| 4 | 2009 | $18,898.50 |
| 4 | 2010 | $20,454.81 |
| 4 | 2011 | $19,864.47 |
| 4 | 2012 | $17,870.09 |
| 4 | 2013 | $17,614.71 |
| 4 | 2014 | $16,715.43 |
| 4 | 2015 | $16,802.66 |
| 4 | 2016 | $15,360.55 |
| 4 | 2017 | $11,105.52 |
| 4 | 2018 | $8,657.55 |
| 4 | 2019 | $9,061.32 |
| 4 | 2020 | $7,937.38 |
| 4 | 2021 | $7,572.84 |
| 5 | 2006 | $28,335.80 |
| 5 | 2007 | $26,366.83 |
| 5 | 2008 | $25,888.44 |
| 5 | 2009 | $29,454.65 |
| 5 | 2010 | $31,849.67 |
| 5 | 2011 | $32,375.78 |
| 5 | 2012 | $30,277.25 |
| 5 | 2013 | $30,682.49 |
| 5 | 2014 | $29,962.04 |
| 5 | 2015 | $27,544.52 |
| 5 | 2016 | $24,622.74 |
| 5 | 2017 | $18,804.44 |
| 5 | 2018 | $16,148.86 |
| 5 | 2019 | $16,116.31 |
| 5 | 2020 | $14,486.36 |
| 5 | 2021 | $15,279.93 |

**eTable 7.** **Annual average costs per patient and region 2006-2021.** E: eastnorth; MW: mid-west; W: west; S: south

| Year | Region | Comorbid HF |
| --- | --- | --- |
| 2006 | E | $8,638.66 |
| 2007 | E | $8,217.42 |
| 2008 | E | $7,814.23 |
| 2009 | E | $8,204.94 |
| 2010 | E | $10,670.59 |
| 2011 | E | $10,736.30 |
| 2012 | E | $11,017.44 |
| 2013 | E | $11,846.78 |
| 2014 | E | $14,134.14 |
| 2015 | E | $13,911.61 |
| 2016 | E | $13,027.61 |
| 2017 | E | $10,249.37 |
| 2018 | E | $10,573.07 |
| 2019 | E | $10,602.81 |
| 2020 | E | $9,262.25 |
| 2021 | E | $9,477.77 |
| 2006 | MW | $8,992.51 |
| 2007 | MW | $9,442.97 |
| 2008 | MW | $9,599.63 |
| 2009 | MW | $10,249.44 |
| 2010 | MW | $10,965.15 |
| 2011 | MW | $11,283.11 |
| 2012 | MW | $11,061.29 |
| 2013 | MW | $11,934.14 |
| 2014 | MW | $10,976.70 |
| 2015 | MW | $11,388.65 |
| 2016 | MW | $10,587.66 |
| 2017 | MW | $10,149.74 |
| 2018 | MW | $9,245.38 |
| 2019 | MW | $9,247.37 |
| 2020 | MW | $8,926.54 |
| 2021 | MW | $9,433.52 |
| 2006 | S | $9,372.37 |
| 2007 | S | $9,123.95 |
| 2008 | S | $9,152.59 |
| 2009 | S | $10,182.20 |
| 2010 | S | $11,297.04 |
| 2011 | S | $12,458.98 |
| 2012 | S | $12,585.57 |
| 2013 | S | $13,701.92 |
| 2014 | S | $14,123.78 |
| 2015 | S | $15,539.83 |
| 2016 | S | $15,074.19 |
| 2017 | S | $8,603.10 |
| 2018 | S | $7,612.46 |
| 2019 | S | $8,362.09 |
| 2020 | S | $6,654.68 |
| 2021 | S | $6,646.12 |
| 2006 | W | $12,255.48 |
| 2007 | W | $10,335.71 |
| 2008 | W | $7,592.10 |
| 2009 | W | $11,162.54 |
| 2010 | W | $11,968.58 |
| 2011 | W | $10,016.45 |
| 2012 | W | $9,280.23 |
| 2013 | W | $10,447.46 |
| 2014 | W | $10,576.97 |
| 2015 | W | $10,766.77 |
| 2016 | W | $11,040.81 |
| 2017 | W | $10,212.31 |
| 2018 | W | $8,662.17 |
| 2019 | W | $8,095.32 |
| 2020 | W | $7,818.03 |
| 2021 | W | $7,377.87 |

**eTable 8.** **The average costs per patient and state over 2006-2021.**

| States | Comorbid HF |
| --- | --- |
| KS | $15,624.27 |
| VT | $14,888.58 |
| MN | $14,696.76 |
| VA | $14,225.05 |
| TX | $13,123.09 |
| SD | $11,821.41 |
| MS | $11,768.09 |
| AZ | $11,740.59 |
| SC | $11,527.01 |
| MT | $11,437.59 |
| IL | $11,381.23 |
| NV | $11,234.27 |
| HI | $11,125.44 |
| NC | $11,075.03 |
| OR | $11,007.57 |
| OK | $10,918.51 |
| MD | $10,917.96 |
| DC | $10,847.99 |
| NY | $10,838.66 |
| NE | $10,728.03 |
| PA | $10,672.66 |
| OH | $10,579.07 |
| TN | $10,434.44 |
| RI | $10,335.56 |
| CO | $10,199.35 |
| FL | $10,176.54 |
| IA | $10,171.41 |
| CA | $10,125.18 |
| WV | $9,970.70 |
| NJ | $9,837.99 |
| MA | $9,807.81 |
| CT | $9,765.48 |
| KY | $9,571.58 |
| IN | $9,358.73 |
| ND | $9,344.85 |
| AL | $9,338.49 |
| WI | $8,797.15 |
| AK | $8,572.54 |
| AR | $8,552.78 |
| WY | $8,479.70 |
| ME | $8,385.80 |
| UT | $8,265.62 |
| LA | $8,234.27 |
| WA | $8,123.36 |
| GA | $8,118.62 |
| MI | $8,115.53 |
| MO | $8,064.80 |
| NM | $7,843.16 |
| DE | $7,622.25 |
| NH | $7,482.90 |
| ID | $6,664.28 |

**eTable 9. Annual average costs per patient and insurance type 2006-2021.**

| Year | Insurance | Comorbid HF category |
| --- | --- | --- |
| 2006 | Commercial | $8,865.32 |
| 2007 | Commercial | $8,630.39 |
| 2008 | Commercial | $8,672.59 |
| 2009 | Commercial | $9,652.05 |
| 2010 | Commercial | $12,292.62 |
| 2011 | Commercial | $13,209.90 |
| 2012 | Commercial | $12,949.91 |
| 2013 | Commercial | $15,343.77 |
| 2014 | Commercial | $15,307.58 |
| 2015 | Commercial | $16,028.48 |
| 2016 | Commercial | $16,670.70 |
| 2017 | Commercial | $21,371.05 |
| 2018 | Commercial | $19,854.64 |
| 2019 | Commercial | $20,566.03 |
| 2020 | Commercial | $19,569.15 |
| 2021 | Commercial | $19,273.59 |
| 2006 | Medicaid | $8,112.72 |
| 2007 | Medicaid | $7,264.16 |
| 2008 | Medicaid | $6,248.33 |
| 2009 | Medicaid | $6,632.25 |
| 2010 | Medicaid | $5,362.56 |
| 2011 | Medicaid | $7,101.83 |
| 2012 | Medicaid | $7,535.11 |
| 2013 | Medicaid | $7,880.33 |
| 2014 | Medicaid | $7,913.30 |
| 2015 | Medicaid | $7,286.17 |
| 2016 | Medicaid | $6,342.03 |
| 2017 | Medicaid | $10,367.22 |
| 2018 | Medicaid | $10,162.24 |
| 2019 | Medicaid | $8,605.42 |
| 2020 | Medicaid | $7,341.37 |
| 2021 | Medicaid | $7,303.25 |
| 2006 | Medicare Risk | $7,255.85 |
| 2007 | Medicare Risk | $7,638.67 |
| 2008 | Medicare Risk | $7,389.51 |
| 2009 | Medicare Risk | $7,634.34 |
| 2010 | Medicare Risk | $8,632.06 |
| 2011 | Medicare Risk | $8,832.19 |
| 2012 | Medicare Risk | $9,034.52 |
| 2013 | Medicare Risk | $9,263.77 |
| 2014 | Medicare Risk | $10,147.56 |
| 2015 | Medicare Risk | $10,153.80 |
| 2016 | Medicare Risk | $10,052.18 |
| 2017 | Medicare Risk | $11,652.07 |
| 2018 | Medicare Risk | $10,648.83 |
| 2019 | Medicare Risk | $10,741.44 |
| 2020 | Medicare Risk | $10,307.85 |
| 2021 | Medicare Risk | $10,833.80 |
| 2006 | Self-Insured | $6,587.78 |
| 2007 | Self-Insured | $8,407.72 |
| 2008 | Self-Insured | $9,444.51 |
| 2009 | Self-Insured | $10,233.69 |
| 2010 | Self-Insured | $11,254.17 |
| 2011 | Self-Insured | $10,894.58 |
| 2012 | Self-Insured | $9,187.74 |
| 2013 | Self-Insured | $9,489.79 |
| 2014 | Self-Insured | $10,830.34 |
| 2015 | Self-Insured | $11,152.20 |
| 2016 | Self-Insured | $7,065.89 |
| 2017 | Self-Insured | $10,490.95 |
| 2006 | Medicare Cost | $11,385.73 |
| 2007 | Medicare Cost | $10,755.29 |
| 2008 | Medicare Cost | $8,778.04 |
| 2009 | Medicare Cost | $11,357.52 |
| 2010 | Medicare Cost | $11,563.00 |
| 2011 | Medicare Cost | $11,069.74 |
| 2012 | Medicare Cost | $11,275.28 |
| 2013 | Medicare Cost | $12,012.79 |
| 2014 | Medicare Cost | $13,146.06 |
| 2015 | Medicare Cost | $13,794.96 |
| 2016 | Medicare Cost | $12,940.40 |
| 2017 | Medicare Cost | $2,447.83 |
| 2018 | Medicare Cost | $2,530.90 |
| 2019 | Medicare Cost | $2,318.26 |
| 2020 | Medicare Cost | $2,221.31 |
| 2021 | Medicare Cost | $1,748.98 |

**eTable 10. Annual average self-payment per patient and payment type 2006-2021.**

| year | Insurance | Comorbid HF |
| --- | --- | --- |
| 2006 | Commercial | $3,522.21 |
| 2007 | Commercial | $3,403.27 |
| 2008 | Commercial | $3,666.49 |
| 2009 | Commercial | $3,751.84 |
| 2010 | Commercial | $4,515.53 |
| 2011 | Commercial | $4,487.85 |
| 2012 | Commercial | $4,186.14 |
| 2013 | Commercial | $4,873.65 |
| 2014 | Commercial | $3,925.87 |
| 2015 | Commercial | $3,321.59 |
| 2016 | Commercial | $3,419.50 |
| 2017 | Commercial | $3,043.70 |
| 2018 | Commercial | $3,160.50 |
| 2019 | Commercial | $3,574.21 |
| 2020 | Commercial | $3,744.57 |
| 2021 | Commercial | $3,336.48 |
| 2006 | Medicaid | $477.08 |
| 2007 | Medicaid | $386.42 |
| 2008 | Medicaid | $848.26 |
| 2009 | Medicaid | $1,239.15 |
| 2010 | Medicaid | $1,102.11 |
| 2011 | Medicaid | $1,760.61 |
| 2012 | Medicaid | $2,253.75 |
| 2013 | Medicaid | $2,474.98 |
| 2014 | Medicaid | $2,684.91 |
| 2015 | Medicaid | $1,982.47 |
| 2016 | Medicaid | $1,013.28 |
| 2017 | Medicaid | $2,819.61 |
| 2018 | Medicaid | $2,302.61 |
| 2019 | Medicaid | $3,511.30 |
| 2020 | Medicaid | $4,324.38 |
| 2021 | Medicaid | $2,227.43 |
| 2006 | Medicare Risk | $441.20 |
| 2007 | Medicare Risk | $787.40 |
| 2008 | Medicare Risk | $645.99 |
| 2009 | Medicare Risk | $693.57 |
| 2010 | Medicare Risk | $664.07 |
| 2011 | Medicare Risk | $572.99 |
| 2012 | Medicare Risk | $562.37 |
| 2013 | Medicare Risk | $697.59 |
| 2014 | Medicare Risk | $938.77 |
| 2015 | Medicare Risk | $1,132.83 |
| 2016 | Medicare Risk | $1,129.63 |
| 2017 | Medicare Risk | $1,656.20 |
| 2018 | Medicare Risk | $1,100.92 |
| 2019 | Medicare Risk | $1,305.11 |
| 2020 | Medicare Risk | $1,027.47 |
| 2021 | Medicare Risk | $1,038.64 |
| 2006 | Self-Insured | $1,177.53 |
| 2007 | Self-Insured | $5,445.35 |
| 2008 | Self-Insured | $6,081.43 |
| 2009 | Self-Insured | $6,627.44 |
| 2010 | Self-Insured | $7,264.40 |
| 2011 | Self-Insured | $6,790.82 |
| 2012 | Self-Insured | $438.87 |
| 2013 | Self-Insured | $507.23 |
| 2014 | Self-Insured | $309.57 |
| 2015 | Self-Insured | $509.27 |
| 2016 | Self-Insured | $278.40 |
| 2017 | Self-Insured | $503.10 |
| 2006 | Medicare Cost | $10,589.26 |
| 2007 | Medicare Cost | $9,848.39 |
| 2008 | Medicare Cost | $7,898.29 |
| 2009 | Medicare Cost | $10,491.22 |
| 2010 | Medicare Cost | $10,690.36 |
| 2011 | Medicare Cost | $10,139.86 |
| 2012 | Medicare Cost | $10,356.42 |
| 2013 | Medicare Cost | $11,044.29 |
| 2014 | Medicare Cost | $12,055.95 |
| 2015 | Medicare Cost | $12,673.62 |
| 2016 | Medicare Cost | $11,774.97 |
| 2017 | Medicare Cost | $1,288.36 |
| 2018 | Medicare Cost | $1,350.51 |
| 2019 | Medicare Cost | $999.29 |
| 2020 | Medicare Cost | $1,041.78 |
| 2021 | Medicare Cost | $488.61 |

**eFigure 1. PharMetrics Plus for Academics Enrollee Characteristics**

**eFigure 2. The annual average costs per patient and age group trends from 2006 to 2021.** Age Group 1: >=85 years, Age Group 2: 80-84 years, Age Group 3: 75-79 years, Age Group 4: 70-74 years, Age Group 5: 65-69 years, Age Group 6: 60-64 years, Age Group 7: 55-59 years, Age Group 8: 50-54 years, and Age Group 9: <50 years.

A)


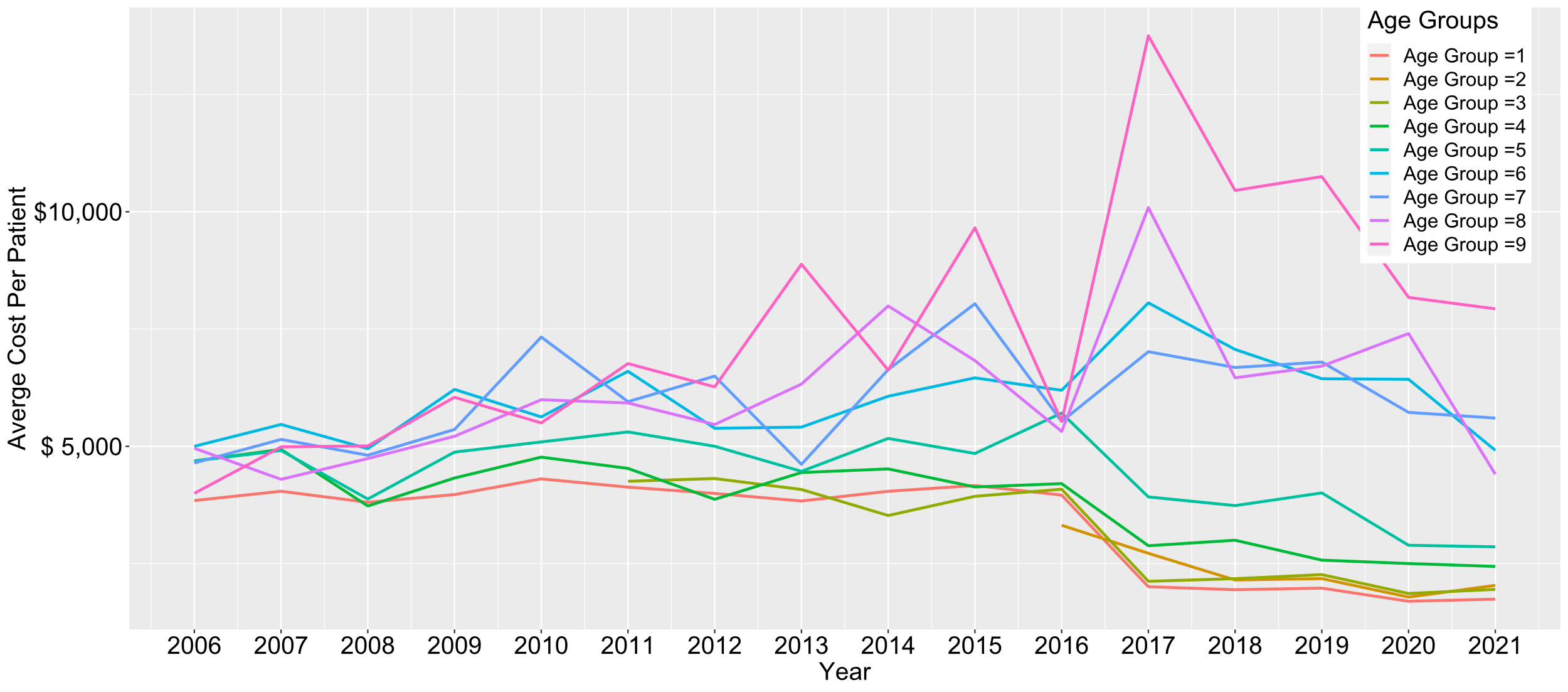


B)

**eFigure 3. The annual average costs per patient and gender trends from 2006 to 2021.**


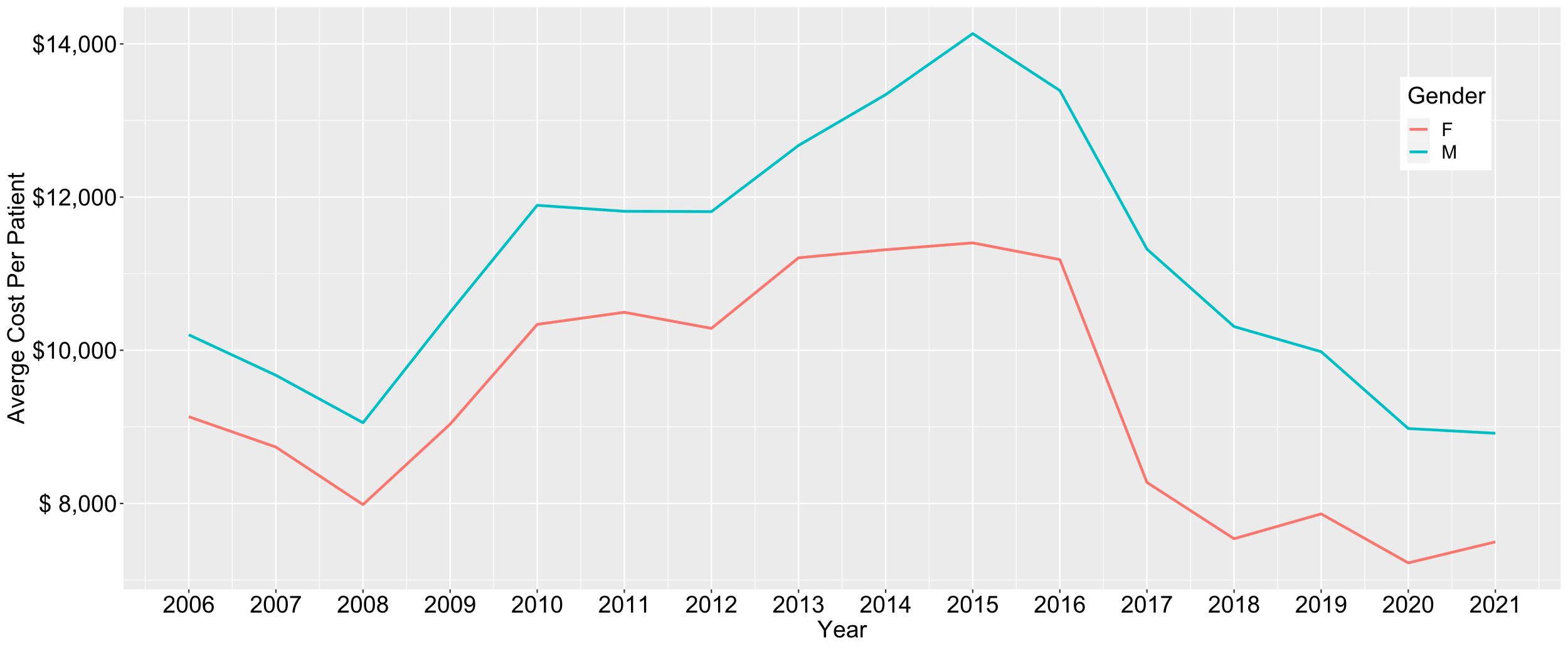


**eFigure 4. The annual average cost per patient and comorbidity index group trends from 2006 to 2021**.

**
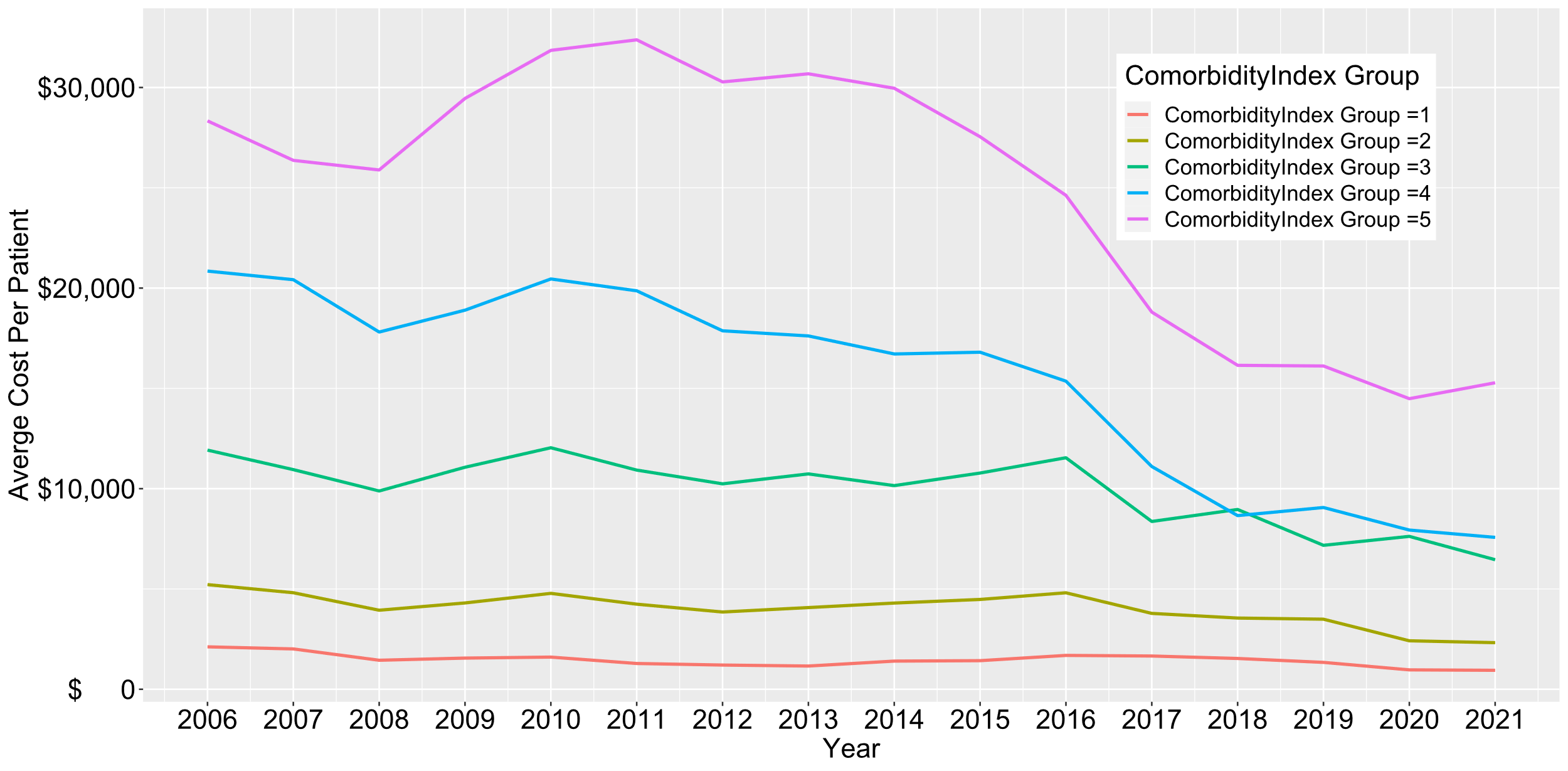
**

**eFigure 5. The annual average medical costs per patient and region trends from 2006-2021.**

**
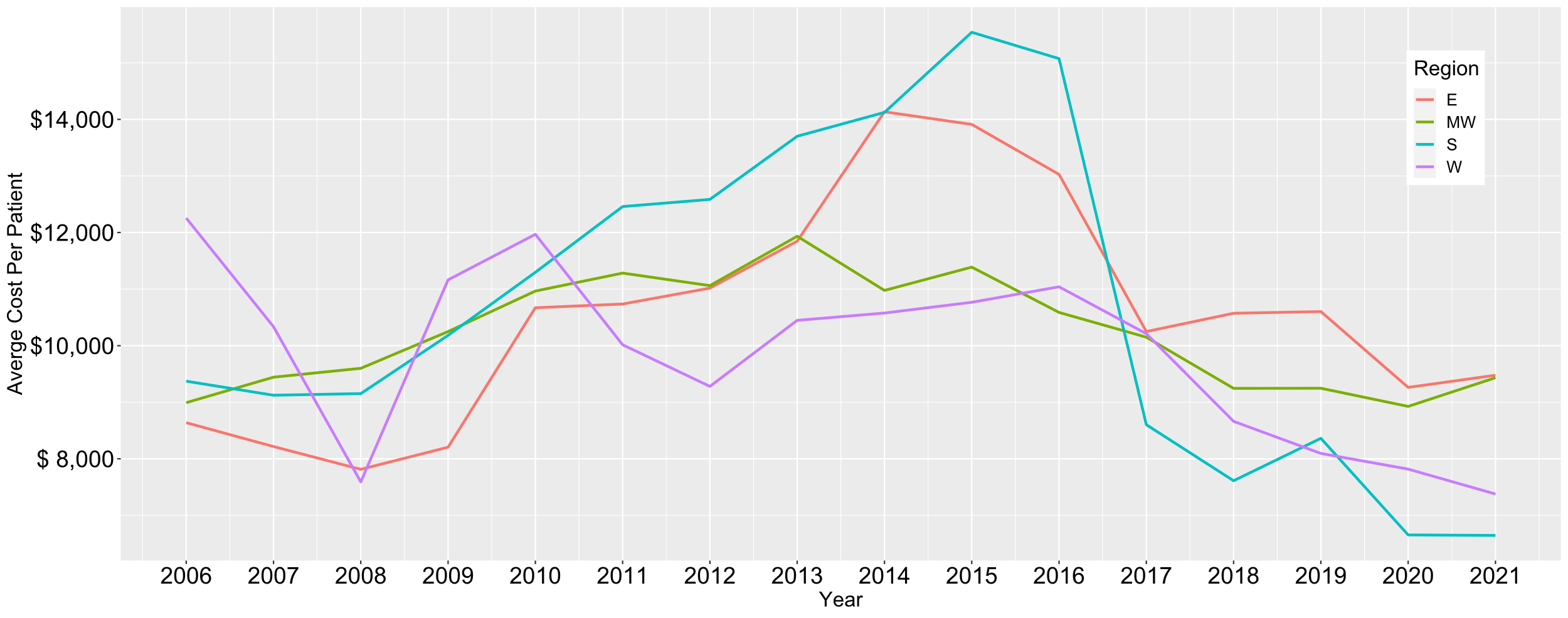
**

**eFigure 6.** **The annual average self-payment per patient and insurance type trends 2006-2021.**


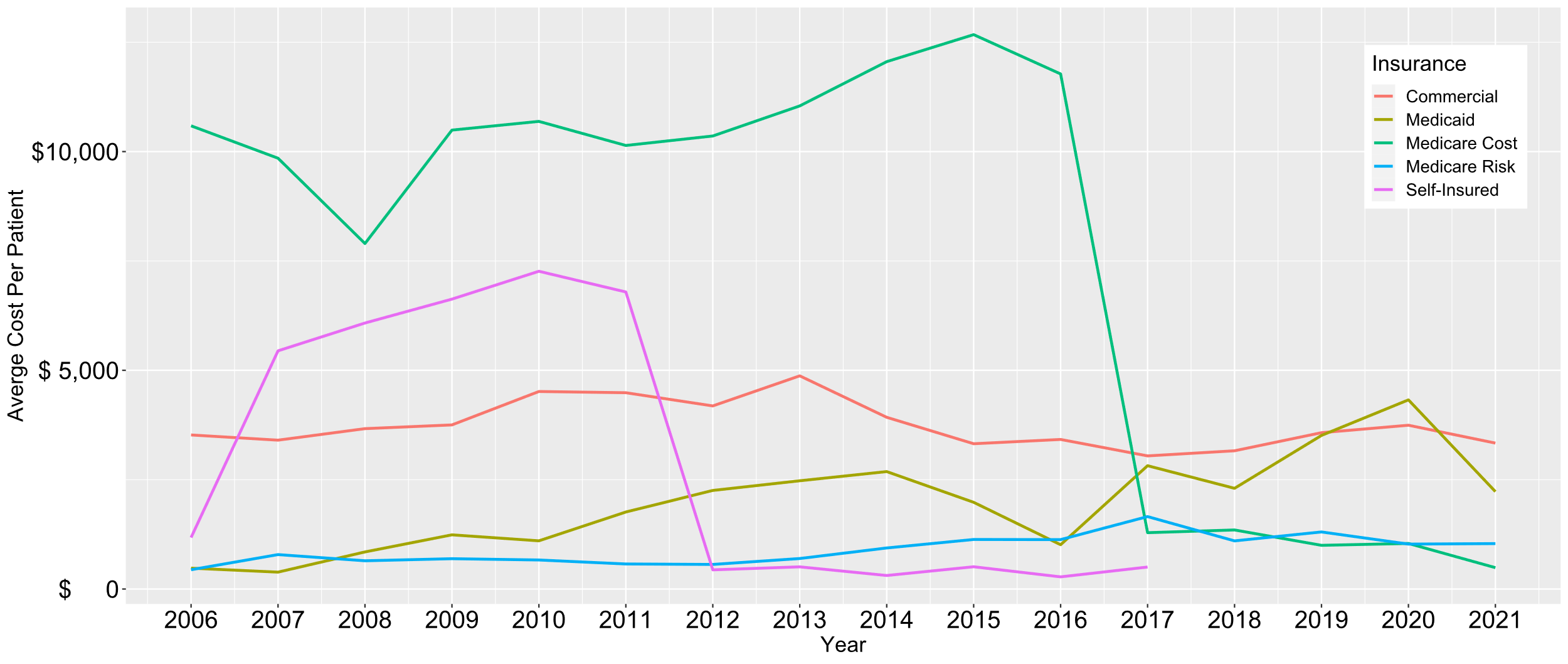

Supplement: Supplementary file 1 — Supplementary Material 1 [file 12913_2024_11240_MOESM1_ESM.docx]
